# Regulatory Mechanism of Nicotine Degradation in *Pseudomonas putida*

**DOI:** 10.1128/mBio.00602-19

**Published:** 2019-06-04

**Authors:** Haiyang Hu, Lijuan Wang, Weiwei Wang, Geng Wu, Fei Tao, Ping Xu, Zixin Deng, Hongzhi Tang

**Affiliations:** a State Key Laboratory of Microbial Metabolism, Joint International Research Laboratory of Metabolic and Developmental Sciences, and School of Life Sciences and Biotechnology, Shanghai Jiao Tong University, Shanghai, People’s Republic of China; Korea Advanced Institute of Science and Technology; University of Applied Sciences Northwestern Switzerland; Institute of Biochemistry and Molecular Biology, Albert-Ludwigs University

**Keywords:** biodegradation, metabolic regulation, nicotinamide, *Pseudomonas*, transcription repressor

## Abstract

Nicotine, a toxic and addictive alkaloid from tobacco, is an environmental pollutant in areas near cigarette production facilities. Over the last decade, our group has studied, in depth, the pyrrolidine pathway of nicotine degradation in Pseudomonas putida S16. However, little is known regarding whole mechanism(s) regulating transcription of the nicotine degradation pathway gene cluster. In the present study, we comprehensively elucidate an overall view of the NicR2-mediated two-step mechanism regulating 3-succinoyl-pyridine (SP) biotransformation, which involves the association of free NicR2 with two promoters and the dissociation of NicR2 from the NicR2-promoter complex. NicR2 can bind to another promoter, *Pspm*, and regulate expression of the nicotine-degrading genes in the middle of *nic2* gene cluster, which are not controlled by the previously reported *Phsp* promoter. We identified the function of the inverted repeat bases on the two promoters responsible for NicR2 binding and found out that the –35/–10 motif for RNA polymerase is overlapped by the NicR2 binding site. We clarify the exact role of 6-hydroxy-3-succinoyl-pyridine (HSP), which acts as an antagonist and may prevent binding of free NicR2 to the promoters but cannot release NicR2 from the promoters. Finally, a regulatory model is proposed, which consists of three parts: the interaction between NicR2 and two promoters (*Pspm* and *Phsp*), the interaction between NicR2 and two effectors (HSP and SP), and the interaction between NicR2 and RNA polymerase.

## Abstract (precis)

We report the entire process underlying the NicR2 regulatory mechanism from association between free NicR2 and two promoters to dissociation of the NicR2-promoter complex. NicR2 can bind to another promoter, *Pspm*, which controls expression of nicotine-degrading genes that are not controlled by the *Phsp* promoter. We identified specific nucleotides of the *Pspm* promoter responsible for NicR2 binding. HSP was further demonstrated as an antagonist, which prevents the binding of NicR2 to the *Pspm* and *Phsp* promoters, by locking NicR2 in the derepression conformation. The competition between NicR2 and RNA polymerase is essential to initiate transcription of nicotine-degrading genes. This study extends our understanding of molecular mechanisms in biodegradation of environmental pollutants and toxicants.

## INTRODUCTION

Transcriptional regulation is a vital and universally required biological phenomenon that enables organisms to efficiently control development, take up nutrients, conserve energy, and compete with other organisms (1). Many proteins and promoter types are known to participate in transcriptional regulatory networks in bacteria, which enable them to respond to changing environmental conditions such as temperature, salinity, and the presence of toxic molecules (2). The regulatory proteins in bacteria can be categorized into at least 20 different families based on DNA-binding motifs (3, 4). With diverse DNA-binding and ligand-binding motifs, these regulatory proteins are involved in a variety of physiological activities. For example, LysR-type regulators negatively regulate their own expression and positively regulate catabolic gene expression in response to effector compounds, while the AraC family XylS regulator controls TOL *meta*-cleavage pathway expression (5). Many known regulators involved in pollutant degradation belong to the TetR family. Examples include 6-hydroxy-d-nicotine oxidase (HdnoR), a gene repressor in response to 6-hydroxy-l-nicotine and 6-hydroxy-d-nicotine (6); AlkX from *Dietzia* sp. strain DQ12-45-1b, in *n*-alkane degradation (7); and NicR2 in the transcription of several genes in the distal region involved in nicotine degradation (8).

**FIG 1:**
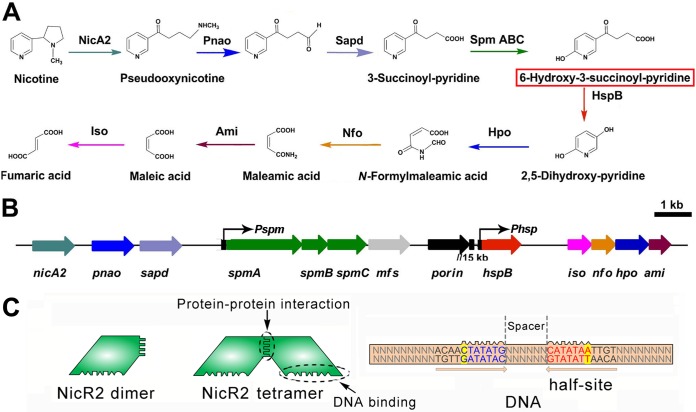
Molecular pathway, its gene cluster, and regulatory model of nicotine degradation in P. putida S16. (A) Catabolic pathway of nicotine in strain S16. The SpmABC enzyme transforms 3-succinoyl-pyridine (SP) into 6-hydroxy-3-succinoyl-pyridine (HSP). HSP is the small molecular effector of NicR2. (B) *nic2* gene cluster for nicotine degradation with the genes in the distal region from *hspB* to *ami.* The *Phsp* promoter is immediately upstream of the *hspB* gene (13). (C) Half-site model of NicR2. Two NicR2 dimers bind to the inverted repeat on the *Phsp* promoter (one dimer binds to one half-site) (8).

Nicotine is not only a toxic and addictive alkaloid from tobacco that harms smokers (9) but also an environmental pollutant (10), especially in areas of tobacco production. Over the last decade, our group has investigated the pyrrolidine pathway of nicotine degradation in Pseudomonas putida S16 in detail (11, 12). Functional nicotine degradation genes of the *nic2* gene cluster were identified (Fig. 1A and B) (13–15). Furthermore, we found a regulator, NicR2, that regulates the expression of several genes in the distal region. It acts via a previously unknown half-site DNA-binding mechanism in the presence of an inverted repeat sequence in the *Phsp* promoter (this repeat contains two half-sites) (Fig. 1C). In addition, we resolved the crystal structure of NicR2-HSP complex (HSP, 6-hydroxy-3-succinoyl-pyridine) (16). Despite these advances made in previous studies on the pyrrolidine pathway, several questions remain unanswered. First and foremost, very little is known about regulatory mechanism(s) that may control transcription of the other degradation genes located in the *nic2* gene cluster. In the present study, we report the entire process underlying the regulation of nicotine degradation in *Pseudomonas*. We performed DNA affinity purification to find the related regulators controlling the genes located in the middle of the *nic2* gene cluster, which was identified as NicR2. In addition, *Pspm* a new promoter binding to NicR2 was identified. We focused on three parts: the interaction between NicR2 and the two promoters *Pspm* and *Phsp*, the interaction between NicR2 and the two effectors HSP and 3-succinoyl-pyridine (SP), and the interaction between NicR2 and RNA polymerase. The bases responsible for the NicR2-promoter association and the homotropic effect between the NicR2 dimers were identified, which had not been reported before. Moreover, we clarified the true role of HSP as an antagonist that may prevent the binding of free NicR2 to the *Pspm* and *Phsp* promoters but could not induce the release of NicR2 bound to the promoters. Given the remarkable function of HSP, we demonstrated the significance of the competition between RNA polymerase and NicR2. Finally, we propose a model of regulation of the expression of nicotine degradation genes.

## RESULTS

**FIG 2:**
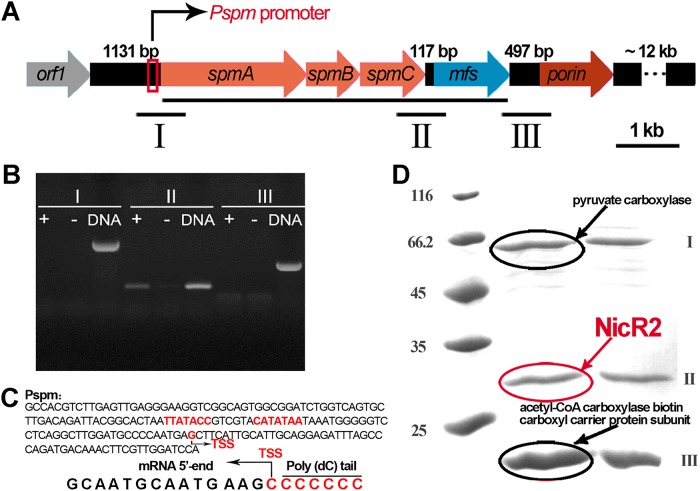
Transcriptional analysis of the middle *nic2* gene cluster. (A) Schematic diagram of the middle *nic2* gene cluster. Six genes (*orf1*, *spmABC*, *mfs*, and *porin*) exist in the middle region of the *nic2* gene cluster. Three fragments contain potential promoters (fragments I, II, and III). (B) Reverse transcription-PCR (RT-PCR) assay. An RT-PCR assay was performed with fragments I, II, and III in both the presence and the absence of nicotine. cDNA was used as positive control. Only fragment II was successfully amplified in the presence of nicotine, indicating that *spmABC* and *mfs* genes were cotranscribed and nicotine-induced. (C) 5′-RACE assay. The figure displays partial sequences of the 5′-RACE products, which are complementary sequences of the bases. The *Pspm* promoter contains a similar inverted repeat (TTATACCGTCGTACATATAA) to that of the *Phsp* promoter (CTATATGTACAGCCATATAA). (D) DNA affinity purification of regulator. Three obvious bands were sequenced. Bands I and III were pyruvate carboxylase and acetyl-CoA carboxylase biotin carboxyl carrier protein subunit, respectively, which were unrelated to the regulation. Band II was identified as NicR2, which could regulate the other promoter, *Phsp*.

**FIG 3:**
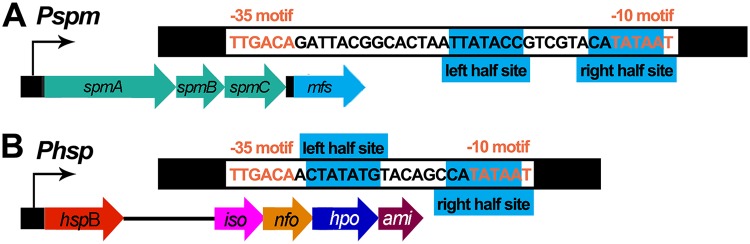
Schematic diagram of *Pspm* and *Phsp*. *Pspm* (A) and *Phsp* (B) promoters have similar molecular structures containing –35/–10 motifs and an inverted repeat. The inverted repeat is completely overlapped on –35/–10 motifs, which leads to direct competition between NicR2 and RNA polymerase.

Since NicR2 regulates gene expression in the distal region of the *nic2* gene cluster (8), we sought to characterize transcriptional regulation of the six genes in the middle region of this cluster (*orf1*, *spmA*, *spmB*, *spmC*, *mfs*, and *porin*) (Fig. 2A). It is known that *spmA*, *spmB*, and *spmC* are polycistronically transcribed (13). In the present study, we found out that *mfs* was also transcribed as a part of this transcriptional unit, and we confirmed that the presence of nicotine increased the transcription of this gene (Fig. 2B). The upstream promoter of this transcriptional unit was annotated as *Pspm*, and its promoter activity was confirmed by conducting the β-galactosidase assay (see Fig. S1 in the supplemental material). The 5′-RACE (5′ rapid amplification of cDNA ends) assay revealed that a G nucleotide at 31 bp at the upstream of the *spmA* start codon is the transcriptional initiation site of the *Pspm* promoter (Fig. 2C). Considering that the middle transcriptional unit was induced by nicotine, we predicted the corresponding regulator as a repressor. We performed DNA affinity purification experiment to find the regulator. The following three proteins were identified: NicR2, pyruvate carboxylase, and an acetyl coenzyme A (acetyl-CoA) carboxylase biotin carboxyl carrier protein subunit (Fig. 2D). Given the action of NicR2 that regulates the transcription of the distal nicotine-degrading genes of the *nic2* gene cluster, it was exciting but logical to find that NicR2 also interacted with the *Pspm* promoter. Similar to the *Phsp* promoter (CTATATGTACAGCCATATAA), there was an inverted repeat on the *Pspm* promoter, located in the vicinity of to the start codon of *spmA*, (TTATACCGTCGTACATATAA) (Fig. 3). We demonstrated the association between NicR2 and promoter *Pspm* using *in vitro* biolayer interferometry and electrophoretic mobility shift assay (i.e., BLI and EMSA) (Fig. S2 and S3).

10.1128/mBio.00602-19.1 FIG S1 The *β-*Galactosidase assay of S16Δ*spmABC*. Mutant S16Δ*spmABC* was employed in this assay, in which *spmABC* was mutated. The strain was cultured in MSM+citrate medium with different compounds (SP or HSP). The *Pspm* promoter is the wild-type. Download FIG S1, TIF file, 1.3 MB. Copyright © 2019 Hu et al. 2019 Hu et al. This content is distributed under the terms of the Creative Commons Attribution 4.0 International license.

10.1128/mBio.00602-19.2 FIG S2 Determination of the association between NicR2 and *Pspm* promoter by biolayer interferometry assay. In phase 1, NicR2 was incubated with the *Pspm* immobilized senor surface in the dilution buffer for 300 s. In phase 2, PBS-Tween buffers were applied to elute the *Pspm*-NicR2 complex from the senor surface for 300 s. Download FIG S2, TIF file, 0.3 MB. Copyright © 2019 Hu et al. 2019 Hu et al. This content is distributed under the terms of the Creative Commons Attribution 4.0 International license.

10.1128/mBio.00602-19.3 FIG S3 Identification of binding site by EMSA. Free, free DNA; Fa, Fa fragment (a 30-bp fragment). Download FIG S3, TIF file, 0.3 MB. Copyright © 2019 Hu et al. 2019 Hu et al. This content is distributed under the terms of the Creative Commons Attribution 4.0 International license.

Regarding the other proteins identified in the DNA affinity purification experiments, pyruvate carboxylase can transform pyruvic acid to oxaloacetic acid, and the acetyl-CoA carboxylase biotin carboxyl carrier protein subunit is a subunit of acetyl-CoA carboxylase. Both of these enzymes have biotin prosthetic groups. Considering that there was a biotin group at the 5′ end of the *Pspm* promoter used for these DNA affinity purification assays, we speculate that these two proteins were purified because they attached to the biotin, not the promoter. Therefore, we predicted that pyruvate carboxylase and an acetyl-CoA carboxylase biotin carboxyl carrier protein subunit are very likely to be unrelated to the regulation.

Similarity in the two inverted repeats led us to suspect that the dual promoter binding capability of NicR2 may be attributed to some specific bases of these inverted repeats. As mentioned above, the binding sites of both *Phsp* and *Pspm* promoters are 20-bp inverted repeats, (CTATATGTACAGCCATATAA) and (TTATACCGTCGTACATATAA), respectively. We named the half-site bases from 5′ to 3′ as left half-site bases (L1, L2, L3, L4, L5, L6, and L7) and right half-site bases (R7, R6, R5, R4, R3, R2, and R1). The right half-sites of both *Pspm* and *Phsp* promoters were strictly conserved (CATATAA), whereas three bases in the left half-site differed between the two promoters. A 6-bp spacer fragment between the half-sites in both promoters provided adequate space for the association between the two dimers since they resided on the promoters and repressed transcription (8).

**FIG 4:**
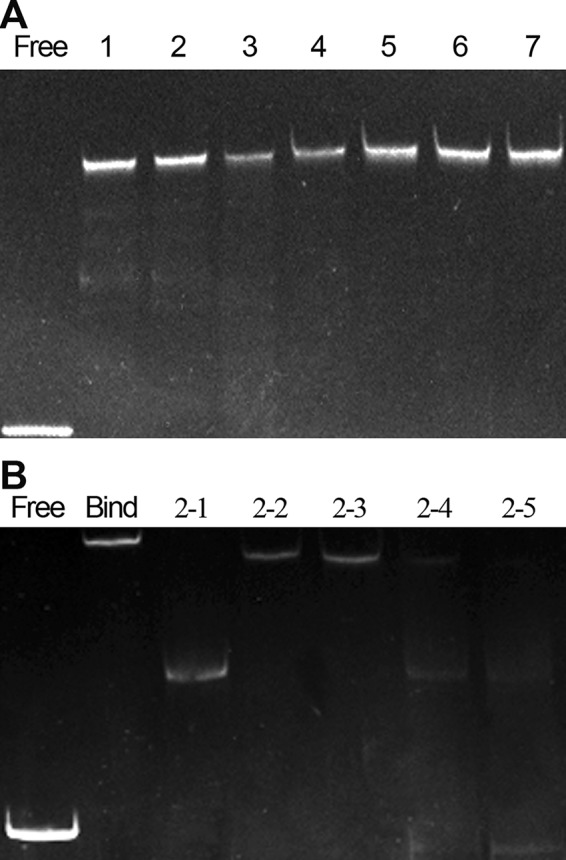
Functional identification of bases on the inverted repeat sequence by EMSA. Each assay consists of a negative control (*Psmp*) and several experimental groups of different mutants. (A) EMSA gel of the single mutants. From left to right: Free, negative control; 1, mutant 1-1; 2, mutant 1-2; 3, mutant 1-3; 4, mutant 1-4; 5, mutant 1-5; 6, mutant 1-6; 7, mutant 1-7. (B) EMSA gel of the double mutants. From left to right: negative control (Free), positive control (Bind), and mutants 2-1, 2-2, 2-3, 2-4, and 2-5.

**FIG 5:**
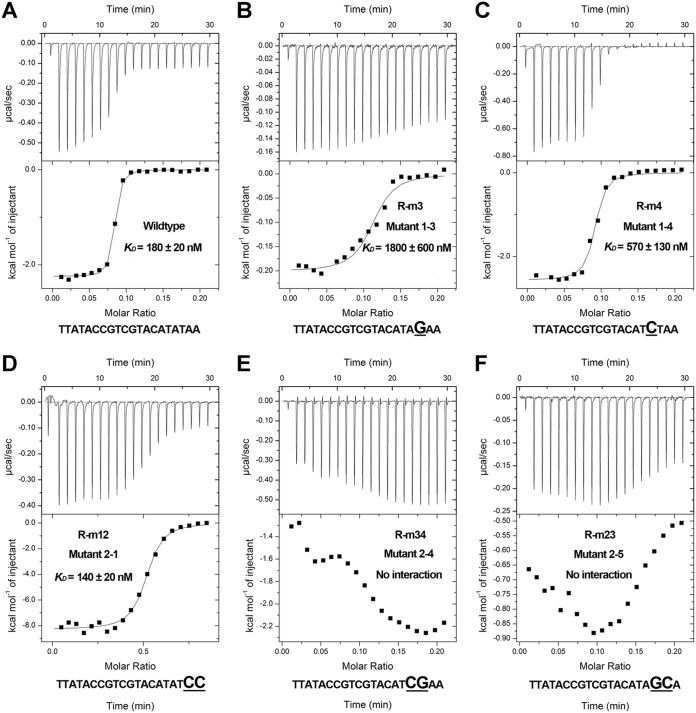
Functional identification of bases on the inverted repeat sequence by ITC. (A) Control group. *K_D_ =* 180 ± 20 nM. (B) Mutant 1-3. *K_D_ =* 1,800 ± 600 nM. (C) Mutant 1-4. *K_D_* *=* 570 ± 130 nM. (D) Mutant 2-1. *K_D_* *=* 140 ± 20 nM. (E and F). Mutants 2-4 and 2-5. No distinct interaction.

We individually mutated each base of the conserved right half-site sequence and used electrophoretic mobility shift assay (EMSA) and isothermal titration calorimetry (ITC) assays to assess the influence of each base on the association between NicR2 and the inverted repeat sequence. The promoter mutants were named R-m1, R-m2, R-m3, R-m4, R-m5, R-m6, and R-m7, respectively (Table S2). Two bands were observed in the wild-type promoter EMSA: a faint nonhomotropic band (a complex of one NicR2 dimer and one DNA fragment) and a bright homotropic band (a complex of two NicR2 dimers and one DNA fragment). One NicR2 dimer associated with one half-site (left or right). Two NicR2 dimers from the right and left half-sites formed an association via a homotropic effect (8). The EMSA binding bands of R-m3 and R-m4 mutants were much weaker than those of the wild type and other mutants, indicating that bases R3 and R4 may be critical for the interaction between NicR2 and the promoters (Fig. 4A). In the ITC assay, the equilibrium dissociation constant (*K_D_*) values of R-m3 and R-m4 mutants were 1,800 ± 600 nM and 570 ± 130 nM, respectively, indicating that R3 had a stronger contribution to the interaction with NicR2 than did R4 (Fig. 5B and C).

10.1128/mBio.00602-19.8 TABLE S2 Mutants applied in EMSA and ITC assays. Download Table S2, XLSX file, 0.01 MB. Copyright © 2019 Hu et al. 2019 Hu et al. This content is distributed under the terms of the Creative Commons Attribution 4.0 International license.

We also synthesized various forms of the promoter with two mutated bases for the right half-site sequence: R-m12, R-m67, R-m45, R-m34, and R-m23 (Table S2). The EMSA showed faint binding bands for R-m34 and R-m23 mutants, indicating that the association between NicR2 and the inverted repeat sequence was disrupted. This suggests the significance of bases R3 and R4 in the association between NicR2 and *Pspm* and *Phsp* promoters. In addition, we still observed a binding band for R-m45, supporting our deduction that R3 contributed more to the association between NicR2 and the two promoters than R4. In addition, the wild-type homotropic band was superseded by a highly enhanced R-m12 mutant nonhomotropic band, indicating that bases R1 and R2 directly participated in the homotropic interaction between the two NicR2 dimers (Fig. 4B). We used the ITC assay to verify our mentioned results (wild type, mutants R-m3, R-m4, R-m12, R-m34, and R-m23). The *K_D_* values of the wild type and mutant R-m12 were 180 ± 20 and 140 ± 20 nM, respectively, indicating that R1 and R2 were essential to the homotropic interaction between the two NicR2 dimers, but not to the association between NicR2 and the promoters. The R-m34 and R-m23 mutants showed no detectable interaction (Fig. 5).

Therefore, we identified the function of the bases on the inverted repeat. The R1 and R2 bases were involved in the homotropic interaction between the two NicR2 dimers. R3 and R4 were essential to the association between NicR2 and the promoters.

**FIG 6:**
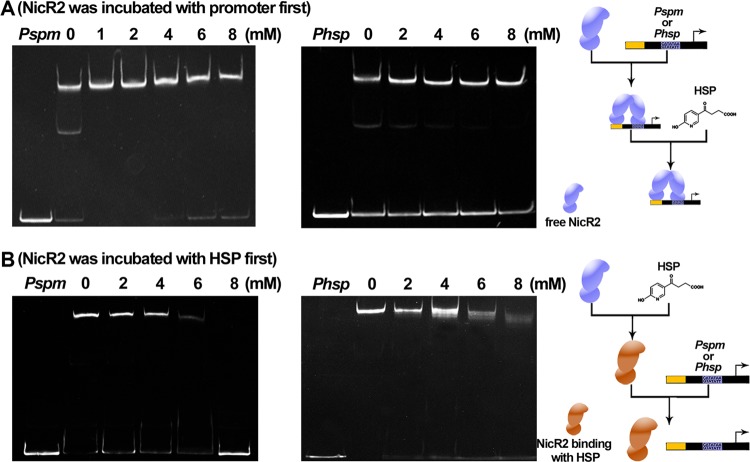
EMSA-based investigation regarding the functional role of HSP. Each assay consisted of a negative control (*Pspm* or *Phsp* only) and several experimental groups in which HSP concentration varied (from 0 to 8 mM). Included DNA fragments consisted of full-length *Pspm* and *Phsp* promoters. (A) When NicR2 was initially incubated with the promoter (i.e., facilitating binding between NicR2 and the *Pspm* or *Phsp* promoter), no concentration of HSP reversed association of NicR2 with the promoters. (B) When NicR2 was initially incubated with HSP (i.e., prior to addition of the promoters), higher concentrations of HSP inhibited association of NicR2 with the promoters.

**FIG 7:**
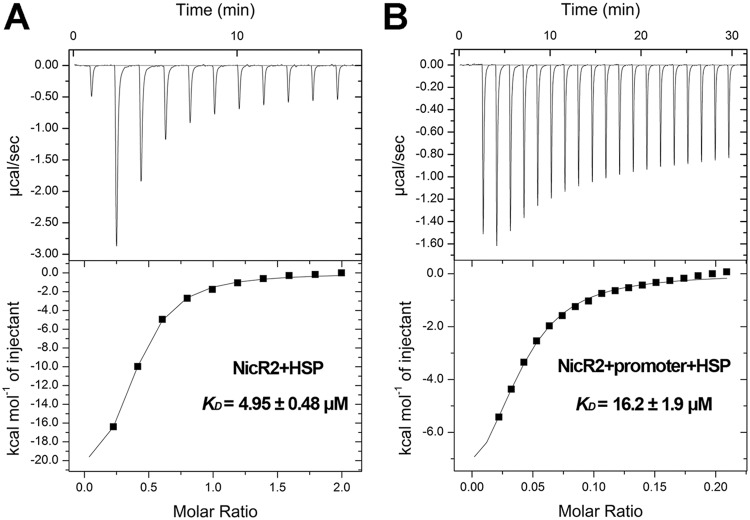
ITC-based investigation regarding the functional role of HSP. (A) Titration of 64 μM NicR2 with 1 mM HSP as the control group. (B) Titration of the mixture of 64 μM NicR2 and 0.1 mM inverted repeat fragment with 1 mM HSP.

In our previous study, we reported that HSP acts as an effector to prevent reassociation between NicR2 and the *Phsp* promoter (8). Thus, EMSA was performed to identify HSP function at both the *Pspm* and the *Phsp* promoters. We accidentally found that sample (DNA fragment, NicR2, and HSP) addition order impacts results, which was missed in previous study. When NicR2 was initially incubated with the *Pspm* or *Phsp* promoter, none of the concentrations of HSP tested was able to cause the dissociation of NicR2-promoter complexes (Fig. 6A). When NicR2 was initially incubated with HSP, prior to the addition of DNA fragment, HSP prevented the association between NicR2 and the two promoters. In addition, HSP completely inhibited the association between NicR2 and the two promoters around at a concentration of approximately 8 mM (Fig. 6B). We performed an ITC assay to assess the energetics of the associations among HSP, NicR2, and the inverted repeat DNA fragment. We used the HSP solution to titrate the NicR2 protein incubated with the inverted repeat DNA fragment and found a *K_D_* value of 16.2 ± 1.9 μM. The association between HSP and free NicR2 was stronger with a *K_D_* value of 4.95 ± 0.48 μM (Fig. 7). Based on the results of EMSA and ITC assays, we inferred that HSP was unable to disrupt the DNA-binding function of NicR2 after association with the *Pspm* and *Phsp* promoters and could only prevent free NicR2 from associating with the *Pspm* and *Phsp* promoters. The *in vivo* assay was performed by detecting the activity of β-galactosidase in the presence or absence of HSP (Fig. S1). The results indicated that HSP activated the *Pspm* promoter, even though it could not directly cause NicR2 displacement from the promoters in the *in vitro* assay.

**FIG 8:**
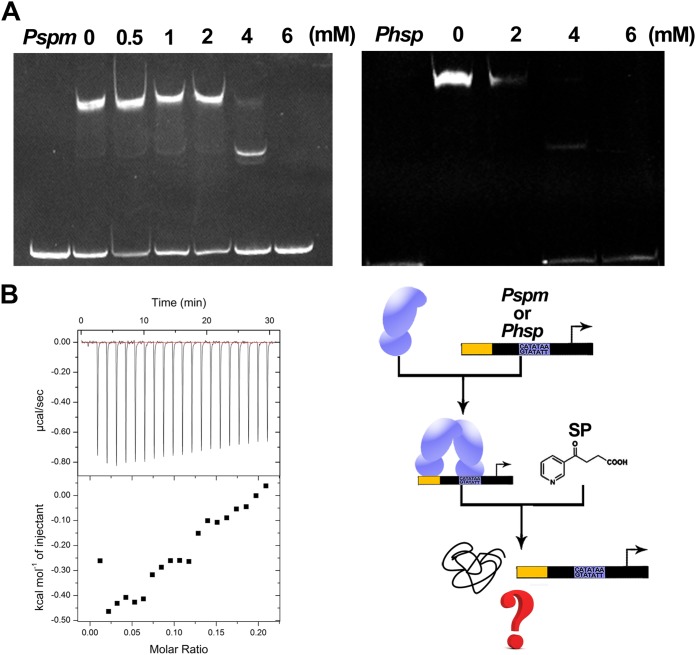
Functional determination of SP. (A) EMSA. The SP concentration increases from 0 to 6 mM. DNA fragments are full-length *Pspm* and *Phsp* promoters. SP derepresses NicR2 *in vitro*. (B) ITC assay. There was no interaction between NicR2 and SP.

One of the genes controlled by the *Phsp* promoter encodes an enzyme that catalyzes the production of HSP. Considering that the *Pspm* promoter is adjacent to the genes encoding an SP-consuming enzyme and that an HSP-*Phsp* interaction exists, we performed an EMSA to identify any potential relationship between SP and two promoters. SP distinctly inhibited the association between NicR2 and the two promoters in the EMSA at 4 mM (Fig. 8). The precipitation of NicR2 was observed by eye after the titration of SP due to the high concentration (64 μM) of NicR2 used in the ITC assay. The EMSA and ITC assays indicated that SP could release NicR2 from the promoters by precipitating NicR2 *in vitro*. However, the effective concentration of SP *in vitro* is 4 mM as mentioned above, which is too high for *in vivo* conditions. Thus, we added 1 mM SP into the β-galactosidase reporter system as we mentioned before, determining whether the lower SP concentration could work *in vivo*. The β-galactosidase activity of SP group is slightly higher than the control group (citrate) (Fig. S1). This indicated that SP was also working at a low concentration *in vivo* but was not as effective as HSP.

The fact that HSP was unable to release NicR2 from the promoters *in vitro* but was able to induce the transcription *in vivo* led us to study how transcription starts. By comparing the whole *Pspm* and *Phsp* sequences, we found other similarities between the two promoters in addition to the inverted repeats. The –35 and –10 motifs of *Pspm* and *Phsp* promoter sequences were identical (TTGACA and TATAAT, respectively). Both motif locations on the promoters were near the transcriptional initiation site, covering the whole inverted repeat (left and right half sites) (Fig. 3). This structure would result in a direct competition between RNA polymerase and NicR2. The competition between RNA polymerase and the repressor is quite common in bacteria (17). However, due to the specific role of HSP, which was unable to release NicR2 from the promoters *in vitro*, this competition is significant for transcriptional initiation in this study. To confirm this competition, we increased the NicR2 concentration in S16 by NicR2-expressing plasmid pME6032-NicR2. Two reconstructed strains, S16_pUCP18k_*Phsp_*GFP and S16_pUCP18k_*Phsp_*GFP/pME6032-NicR2, were used. The value of the fluorescence-intensity (FI)/optical density at 600 nm (OD_600_) ratio in arbitrary units (AU) of S16_pUCP18k_*Phsp_*GFP/pME6032-NicR2 was lower than that of S16_pUCP18k_*Phsp_*GFP (Fig. S4). We also simulated this competition by detecting the promoter activity at different NicR2 concentrations. Strain DH5α-pUCP18K_*Phsp*_GFP/pETDuet_NicR2 was reconstructed to express NicR2 in the presence of IPTG (isopropyl-β-d-thiogalactopyranoside). To inhibit the leaky expression from pETDuet_NicR2, we added 1 g/liter glucose into the first group, of which the AU value of FI/OD_600_ was much higher than those for the other two groups. We constructed another strain DH5α-pUCP18K_*Phsp*_GFP to detect the influence of glucose and IPTG to the promoter activity in the absence of NicR2. The AU values of FI/OD_600_ of DH5α-pUCP18K_*Phsp*_GFP were all around 7,800 with glucose and IPTG, which means that the fluorescence signal of this strain is unrelated to the glucose and IPTG (Fig. S5). All of these results suggest that the increase of NicR2 concentration would lead to the decrease of RNA polymerase competitiveness and reduce the promoter activity. In conclusion, in the absence of HSP, an equilibrium situation occurred, leading to leaky expression of the enzymes controlled by the *Pspm* and *Phsp* promoters. However, in the presence of HSP, the HSP would prevent free NicR2 binding to the promoters. This weakens the competitive edge of NicR2, allowing RNA polymerase to replace the former NicR2 and initiate the transcription.

10.1128/mBio.00602-19.4 FIG S4 Detection of *Phsp* promoter activity at different NicR2 concentrations. Two reconstructed strains were employed, S16_pUCP18k_*Phsp_*GFP (negative control) and S16_pUCP18k_*Phsp_*GFP/pME6032-NicR2 (with plasmid expressing NicR2). S16_pUCP18k_*Phsp_*GFP/pME6032-NicR2 was used to detect *Phsp* promoter activity at a higher NicR2 concentration in strain S16. Download FIG S4, TIF file, 1.3 MB. Copyright © 2019 Hu et al. 2019 Hu et al. This content is distributed under the terms of the Creative Commons Attribution 4.0 International license.

10.1128/mBio.00602-19.5 FIG S5 Detection of *Phsp* promoter activity at different NicR2 concentrations. Two reconstructed strains were used, DH5α-pUCP18K_*Phsp*_GFP (negative control) and DH5α-pUCP18K_*Phsp*_GFP/pETDuet_NicR2 (with plasmid expressing NicR2). Strain DH5α-pUCP18K_*Phsp*_GFP was constructed for the absence of NicR2. Each strain was detected at 1 g/liter glucose and at 0 and 0.2 mM IPTG, respectively. Download FIG S5, TIF file, 1.3 MB. Copyright © 2019 Hu et al. 2019 Hu et al. This content is distributed under the terms of the Creative Commons Attribution 4.0 International license.

## DISCUSSION

Many studies have reported that the intermediates are able to inhibit the repressors, responsible for the corresponding gene cluster(s). For example, in the nicotine acid degradation pathway, which overlaps in part with nicotine degradation along the pyrrolidine pathway, NiaR and BpsR can be inhibited by nicotine acid or its intermediate (18, 19). However, in this study, HSP could only prevent the NicR2 to bind to the promoters, which is different from the reported repressors in nicotine acid degradation. Thus, we compared the crystal structures of several TetR-type proteins with that of NicR2. The DNA binding domain is highly conserved in the TetR protein family. However, these proteins contain a nonconserved ligand-binding domain for specific effectors. In the TetR family, ligand-induced conformational changes always accompany center-to-center distance alterations between their DNA-binding domains. For example, the center-to-center distance between the α3 and α3′ helices of QacR exhibits an increase of 12 Å upon ligand binding (20). These conformational changes make them unsuitable for griping the adjacent position of the major groove (21). However, the center-to-center distance between the α3 and α3′ helices of native NicR2 is 41.3 Å, and the repetitive distance between two successive positions of one major groove is approximately 34 Å (16). This suggests that free NicR2 is unsuitable to bind with the promoters. However, the conformation of NicR2 bound and unbound with HSP shows little difference (Fig. S6). The conformational data cannot explain how NicR2 binds to the promoter and releases from it, which is an unsolved question in the previous study. In this study, we relate the function with the conformation. Free NicR2 is in a natural derepressing conformation, which is unable to hook the right position on the promoters. While HSP binds to NicR2, HSP locks the derepressing conformation and keeps NicR2-HSP complex unable to bind to the promoters, as reported for the ActR ligand-binding conformation (22). This makes HSP able to prevent NicR2 to bind to the promoter but unable to release NicR2 from the promoters. Moreover, in the ITC assay, we observed a heat change in the NicR2+promoter+HSP group (*K_D_* = 16.2 ± 1.9 μM), but it was not as strong as that for the free NicR2+HSP group (*K_D_* = 4.95 ± 0.48 μM) (Fig. 7). This indicates that HSP can bind to NicR2 that has interacted with the promoters but cannot release the DNA-binding domain from the major groove of the inverted repeat. In the TmoS/TmoT regulatory system, some ligands are able to inhibit TmoS autophosphorylation, resulting in their incapacity to stimulate gene expression *in vivo*. The Krell laboratory claims this kind of ligand are antagonists (23), similar to HSP. Thus, we decided to quote this claim here. In addition, this ability requires an appropriate concentration, which means that HSP could not completely prevent NicR2 from the promoters unless the HSP concentration is sufficient to transfer all the NicR2 to the NicR2-HSP complex. In an EMSA, the concentration of NicR2 was much higher than that *in vivo*, leading to the requirement for high concentration of HSP.

10.1128/mBio.00602-19.6 FIG S6 In a previous study, structural comparison of the HSP-bound form (green) and the ligand-free form (magenta) of NicR2 reveals little conformational change (16). Download FIG S6, TIF file, 0.9 MB. Copyright © 2019 Hu et al. 2019 Hu et al. This content is distributed under the terms of the Creative Commons Attribution 4.0 International license.

In addition, we found another effector, SP, which shows a weaker ability to induce the transcription *in vivo* compared to HSP (Fig. S1). SP is able to release NicR2 from the promoters by precipitating NicR2 *in vivo*, which also makes us unable to get the conformation of SP-NicR2 complex. We inferred that the SP concentration *in vivo* is much lower than that *in vitro*, meaning that SP could not fully function *in vivo*. In addition to the low concentration of SP *in vivo*, the NicR2 conformation also causes this ability difference between HSP and SP. HSP contains an additional hydroxyl group compared to SP (16). The absence of a hydroxyl group eliminates the hydrogen bonds donated by R91 and Q118 and makes SP unstable to bind the pocket of NicR2 (Fig. S6). Therefore, due to the low SP concentration *in vivo* and the NicR2 conformation, HSP contributes more to the transcriptional initiation *in vivo* than does SP.

**FIG 9:**
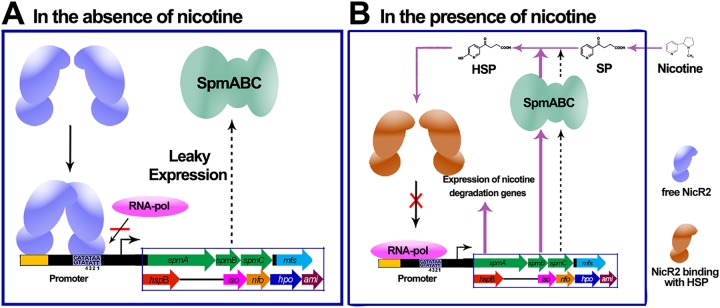
Overall view of the proposed regulatory model shows the integrated process in terms of NicR2 regulatory mechanism, from association between free NicR2 and the two promoters (A, in the absence of nicotine) to dissociation of the NicR2-promoter complex (B, in the presence of nicotine). (A) In the absence of nicotine, free NicR2 (blue) is able to bind to the promoters. Two NicR2 dimers are recruited by the inverted repeat sequence, and the NicR2-promoter complex inhibits the transcription of nicotine-degrading genes. At this stage, the bases R3 (T) and R4 (A) are responsible for the association between NicR2 and the *Pspm* and *Phsp* promoters, and the bases R1 (A) and R2 (A) are responsible for the homotropic effect between the two NicR2 dimers. Leaky gene expression was observed, especially for the expressed SpmABC enzyme (green). (B) In the presence of nicotine, the constitutively expressed SpmABC transforms part of SP into HSP. HSP locks free NicR2 in the derepressing conformation and prevents recombination between free NicR2 and the promoters. RNA polymerase binds to both *Pspm* and *Phsp* promoters and initiates the transcription of nicotine-degrading genes.

Our previous studies on the pyrrolidine pathway of nicotine degradation in P. putida S16, together with this present study, reveal the entire process underlying the NicR2 regulatory mechanism from association between free NicR2 and the two promoters, inhibiting the transcription of nicotine-degrading genes (Fig. 9A), to dissociation of the NicR2-promoter complex, allowing RNA polymerase to initiate transcription (Fig. 9B) (8). In the absence of nicotine, free NicR2 is able to bind to the promoters. Two NicR2 dimers are recruited by the inverted repeat sequence, and the transcription of nicotine-degrading genes is inhibited. In this stage, the bases R3 (T) and R4 (A) are essential for the binding between NicR2 and the *Pspm* and *Phsp* promoters, and the bases R1 (A) and R2 (A) are responsible for the homotropic effect between the two NicR2 dimers.

In the presence of nicotine, the background cellular population of the SpmABC enzyme is sufficient to catalyze the reaction of SP into HSP. HSP locks free NicR2 in a derepressing conformation and prevents any reassociation between NicR2 and the promoters, disturbing the competitive equilibrium between NicR2 and RNA polymerase. Both the *Pspm* and the *Phsp* promoters are exposed to RNA polymerase, and transcription of the genes of the degradation cluster is activated. The HspB enzyme is one of the transcriptional products of the cluster and catalyzes the reaction of HSP into 2,5-dihydroxy-pyridine. This decreases the concentration of HSP and eventually allows transcription to resume inhibition by NicR2 (Fig. 9B).

In conclusion, the regulatory model was summarized and proposed (Fig. 9), which consists of (i) the interaction between NicR2 dimers and the *Pspm* and *Phsp* promoters, (ii) the interaction between NicR2 and effectors HSP and SP, and (iii) the interaction between NicR2 and RNA polymerase. The model reveals an integrated process from association between free NicR2 and two promoters to dissociation of the NicR2-promoter complex. This study offers an overall view of the regulatory mechanism of nicotine degradation in *Pseudomonas* and enriches our understanding of molecular mechanisms in biodegradation of environmental pollutants and toxicants.

## MATERIALS AND METHODS

### Materials.

l-(–)-Nicotine (≥ 99% purity) was obtained from Fluka Chemie GmbH (Switzerland). 3-Succinoyl-pyridine (SP) was purchased from Toronto Research Chemicals (Canada). 6-Hydroxy-3-succinoyl-pyridine (HSP) was purified via a previously described protocol (24). All other reagents and solvents used in this study were of analytical grade and are readily available.

### Semiquantitative reverse transcription-PCR.

Pseudomonas putida S16 (DSM 28022) was cultured overnight in citrate or nicotine medium, and total RNA was extracted using an RNAprep pure cell/bacteria kit (Tiangen Biotech, China), as previously described (25). Genomic DNA of P. putida S16 was used as the positive-control group template.

### Biolayer interferometry.

DNA-protein binding kinetics were measured using the Octet RED96 System (ForteBio). In the preparatory stage, 5′-biotin-TEG-labeled duplex oligonucleotide probes representing the A or G alleles of rs7279549 were immobilized on streptavidin-modified senor surfaces in DNA solutions at a fixed concentration (1 μM). In phase 1, protein interacted with the DNA immobilized senor surface in the dilution buffer for 300 s. In phase 2, phosphate-buffered saline/Tween (PBS-Tween) buffers were used to elute the DNA-protein compound from the senor surface. This phase was sustained for 300 s.

### Determination of the transcriptional start sites.

The transcriptional start sites of the *nic2* gene cluster were identified using a 5′-RACE system (Invitrogen). First-strand cDNA was amplified by the spm-GSP1 primer, after which terminal transferase and dCTP were applied in the tailing treatment of cDNA. The dC-tailed cDNA was amplified using the abridged anchor primer (AAP) and spm-GSP2, and a nested PCR with AAP and primer spm-GSP3 was performed using this PCR product as the template. This amplification product was then cloned into the pMD18-T vector (TaKaRa, Japan) for confirmation by sequencing. All assay primers are shown in Table S1.

10.1128/mBio.00602-19.7 TABLE S1 Primers used in this study. Download Table S1, XLSX file, 0.01 MB. Copyright © 2019 Hu et al. 2019 Hu et al. This content is distributed under the terms of the Creative Commons Attribution 4.0 International license.

### Activity determination of the *Pspm* promoter.

P. putida strain S16△*spmABC*-pME6522-*Pspm* was cultured in citrate, citrate with the addition of 1 mM SP, and citrate with the addition of 1 mM HSP, respectively. *O*-Nitrophenyl-*β*-d-galactopyranoside was added during the *β*-galactosidase activity assay. Data were normalized to the OD_600_ and are expressed in Miller units (26).

### DNA affinity analysis and purification of regulators.

The promoter *Pspm* was commercially modified with biotin at its 5′ end and immobilized on streptavidin beads (Invitrogen). P. putida S16 was cultured overnight in citrate medium, and the harvested cells were resuspended with PBS and disrupted by sonication in an ice-water bath. The insoluble material was removed by centrifugation (12,000 × *g* for 30 min). The crude enzyme preparation and the treated beads were then mixed, followed by incubation at room temperature for 30 min. Magnets were used to collect the beads, and protein was released from the beads by boiling in a water bath for 5 min. SDS-PAGE was performed for the detection of the sample using a 12% gel in a MiniProtean III electrophoresis cell (Bio–Rad). The single bands were cut and characterized by matrix-assisted laser desorption ionization--time of flight mass spectrometry.

### Association between NicR2 and the promoters *in vitro*.

EMSA was performed as previously described (27). All DNA fragments used in EMSAs were amplified from P. putida S16 genomic DNA. Protein NicR2 was purified as previously described (8). The standard 20-μl reaction system contained a 27 nM concentration of a given DNA fragment, 180 nM NicR2, and reaction buffer (10 mM Tris-HCl and 100 mM NaCl). The length of inverted repeat is only about 30 bp, which is unsuitable for operation. Therefore, a 131-bp DNA fragment from the *spmA* gene, which was proved unable to interact with NicR2, was fused to the inverted repeat fragment. The new fragment was 161 bp, which was much easier for operation. The mixture was incubated for 30 min at room temperature, loaded onto a 9% native polyacrylamide gel prepared using 1 M Tris-HCl (pH 8.8), and electrophoresed at 170 V (constant voltage) for 50 min in an ice bath. The gel was stained immediately with SYBR green I according to the manufacturer’s instructions (SBS Genetech, China).

SP and HSP were tested as potential small molecule effectors by EMSA. Two different methods were applied: the first method involved incubation of NicR2 with the putative effector for 15 min at room temperature prior to DNA fragment addition and a 30-min incubation, and the second method involved mixing the DNA fragment and NicR2 prior to the addition of an effector.

### Isothermal titration calorimetry.

DNA fragments and NicR2 were prepared in PBS buffer just before the ITC assay. All DNA fragments, including the inverted repeat regulatory sequence (5′-CACTAAAGCGCCCGTCGTACATATAATAAA-3′), were 30 bp in length. All samples were degassed with vacuum aspiration for five min before analysis with an ITC_200_ instrument (MicroCal). The reaction cell was filled with 64 μM NicR2 solution, and the titration was performed with an initial 0.4-μl injection of 100 μM a given DNA fragment, followed by 19 injections of 2 μl of the DNA fragment spaced at 2-min intervals. Titrating buffer was used as the control. The binding stoichiometry (*N*) value and the equilibrium dissociation constant (*K_D_*) were calculated using Origin 7.0 software.

### Determination of the promoter activity at different NicR2 concentrations.

pUCP18K_*Phsp*_GFP is a GFP-reporter plasmid. pETDuet_NicR2 and pME6032_NicR2 are NicR2 expression plasmids which can be induced by IPTG. Two reconstructed strains were used to detect the promoter activity at different NicR2 concentration in strain S16, S16_pUCP18k_*Phsp_*GFP and S16_pUCP18k_*Phsp_*GFP/pME6032-NicR2. S16_pUCP18k_*Phsp_*GFP is the negative control. The strains were cultured at 30°C for 12 h with suitable antibiotics and 0.2 mM IPTG.

Two reconstructed strains, DH5α- pUCP18K_*Phsp*_GFP and DH5α- pUCP18K_*Phsp*_GFP/pETDuet_NicR2, were used to detect the promoter activity at different NicR2 concentrations in strain DH5α. Each strain was cultured with different IPTG concentrations, i.e., 0, 0.2, and 1 mM, respectively. The strains were cultured at 30°C for 12 h with suitable antibiotics. Fluorescence signal and OD_600_ values were detected by using an ENSPIRE 2300 multimode plate reader (Perkin-Elmer, USA).

10.1128/mBio.00602-19.9 TABLE S3 Function identification of the bases on the IR by ITC. Download Table S3, XLSX file, 0.01 MB. Copyright © 2019 Hu et al. 2019 Hu et al. This content is distributed under the terms of the Creative Commons Attribution 4.0 International license.
