# Supplementary figures and images for: Regulatory Mechanism of Nicotine Degradation in Pseudomonas putida
Source: mBio. 2019 Jun 4;10(3):e00602-19. doi: 10.1128/mBio.00602-19 (PMC6550519; doi:10.1128/mBio.00602-19)

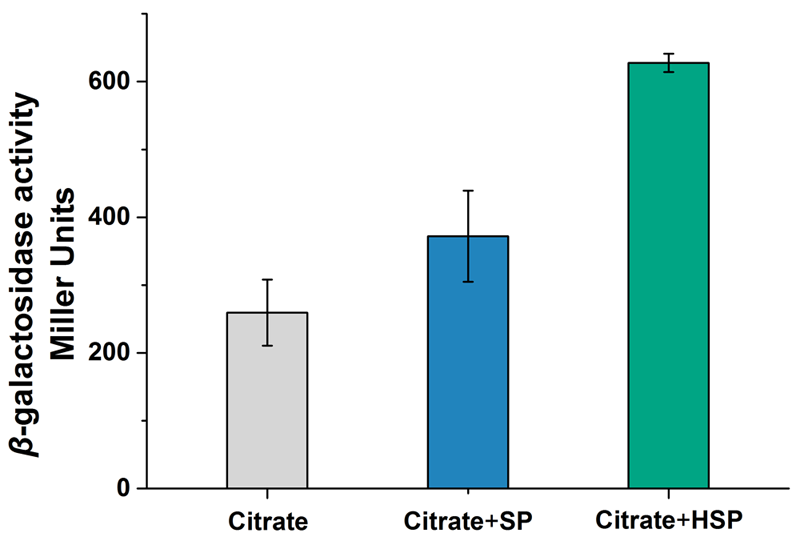

Supplement: FIG S1 [file mBio.00602-19-sf001.tif]

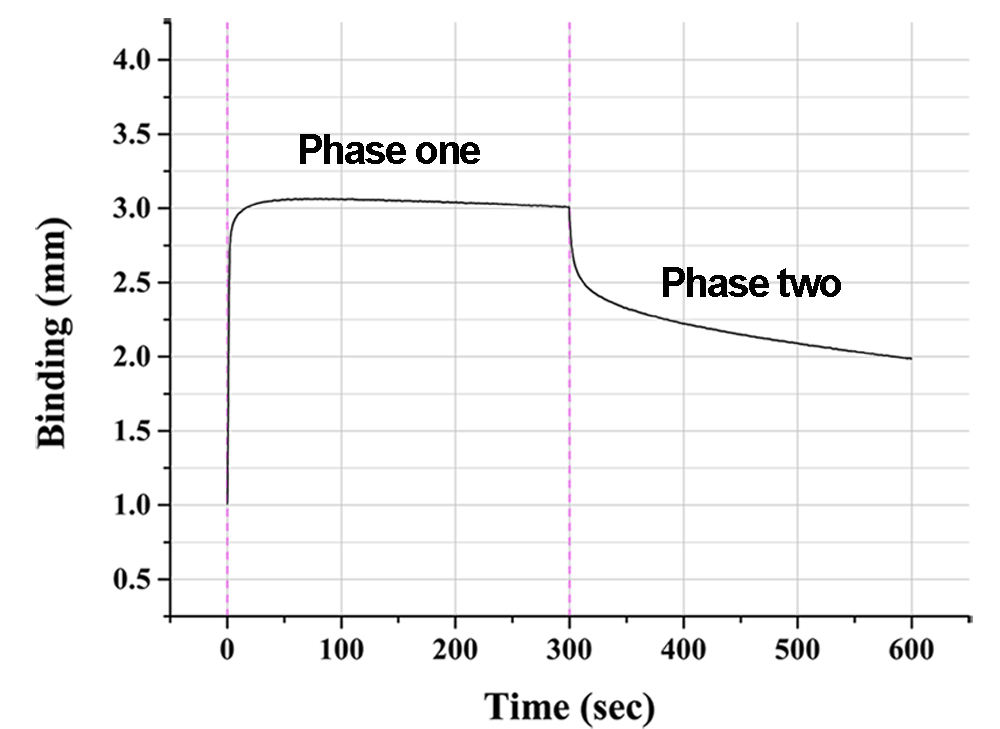

Supplement: FIG S2 [file mBio.00602-19-sf002.tif]

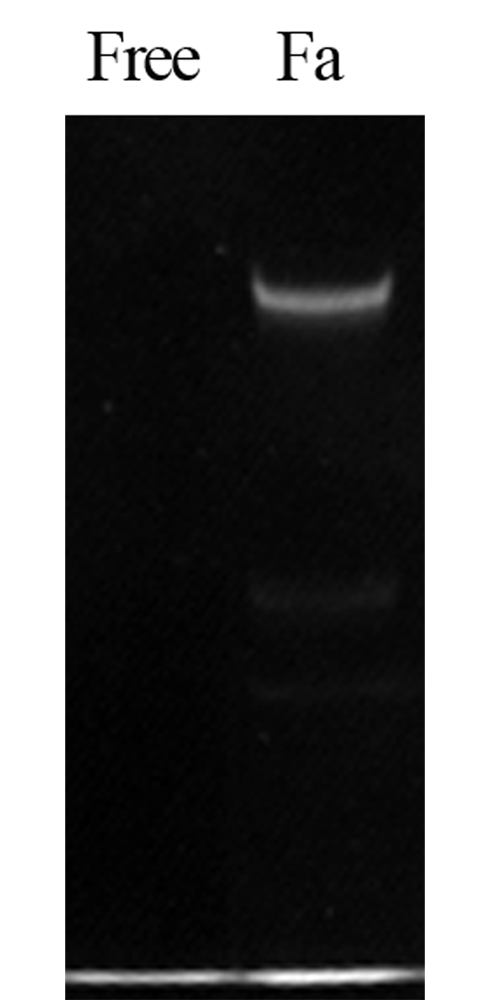

Supplement: FIG S3 [file mBio.00602-19-sf003.tif]

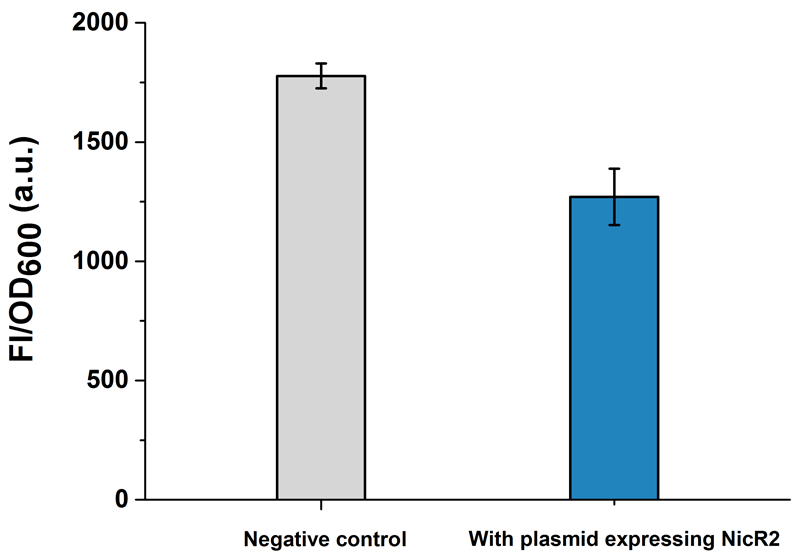

Supplement: FIG S4 [file mBio.00602-19-sf004.tif]

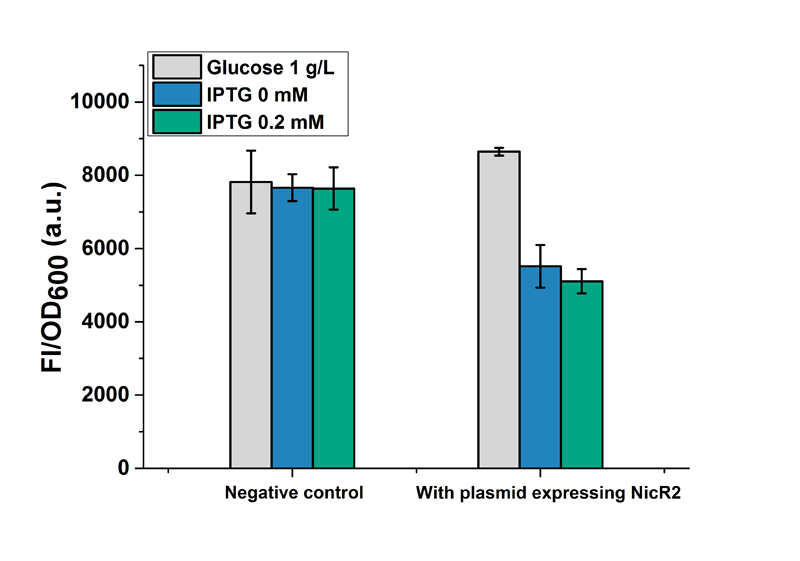

Supplement: FIG S5 [file mBio.00602-19-sf005.tif]

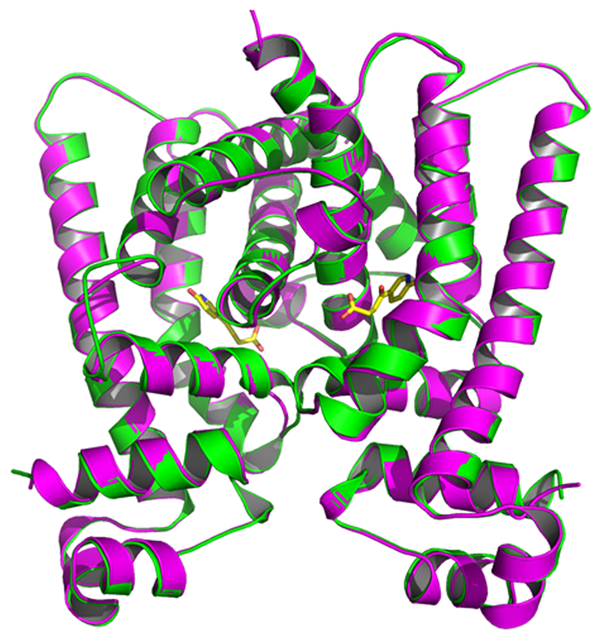

Supplement: FIG S6 [file mBio.00602-19-sf006.tif]
